# Dynamin regulates PLK-1 localization and spindle pole assembly during mitosis

**DOI:** 10.1101/2025.07.21.665896

**Published:** 2025-07-21

**Authors:** Carter Dierlam, Livinus Anyanwu, Stephanie Held, Robert H. Newman, Jyoti Iyer

**Affiliations:** 1 Department of Cell Biology and Molecular Genetics, University of Maryland College Park, College Park, MD 20742; 2 Department of Chemistry and Biochemistry, University of Tulsa, Tulsa, OK 74104; 3 Department of Biology, North Carolina Agricultural and Technical State University, Greensboro, NC 27411.

**Keywords:** dynamin, Plk1, mitosis, midbody, cytokinesis

## Abstract

Accurate cytokinesis is essential for maintaining genomic integrity. Although the GTPase dynamin has been well studied for its role in vesicular trafficking, its function during mitosis remains poorly understood. In this study, we uncover a novel role for the *C. elegans* dynamin homolog, DYN-1, in regulating mitotic spindle pole assembly and the spatiotemporal localization of the key mitotic kinase Polo-like kinase 1 (PLK-1). Our studies demonstrate that the depletion of DYN-1 leads to enlarged metaphase spindle poles and elevated levels of centrosome-associated PLK-1. Strikingly, PLK-1 fails to re-localize from centrosomes to the midbody during late mitosis in a subset of DYN-1–depleted embryos, correlating with abnormal PLK-1 localization at the midbody and defective midbody formation. Importantly, this phenotype is likely not due to increased total PLK-1 protein levels, as DNM2 (human homolog of DYN-1) depletion in HeLa cells did not alter total Plk1 abundance. Together, our findings identify DYN-1 as a new regulator of PLK-1 localization during mitosis and suggest that failure to remove PLK-1 from centrosomes may underlie cytokinesis defects that are observed upon DYN-1 depletion.

## INTRODUCTION

Proper cytokinesis is a prerequisite for the accurate partitioning of the chromosomes to yield two genetically identical daughter cells. Cytokinesis defects can lead to aneuploidy, which is a characteristic feature of cancer cells. Therefore, it is essential to understand the molecular mechanisms governing cytokinesis. The GTPase dynamin is a large ~100 kDa protein that is best known for its role in the scission of endocytic vesicles (Hinshaw JE 2000; Menon et al., 2013; Khurana et al., 2023). The dynamin protein is strongly conserved between humans and the multicellular eukaryotic nematode *Caenorhabditis elegans* (*C. elegans*). *C. elegans* have a single classical dynamin isoform called DYN-1. There is over 60% identity at the amino acid level between human DNM2 and *C. elegans* DYN-1 (Clustal Omega). Interestingly, some of the earliest studies identified dynamin as a microtubule-binding protein (Shpetner and Vallee, 1989). However, dynamin’s function in microtubule-associated processes is less well understood. Studies in various organisms including *C. elegans* and human cells have revealed that dynamin is required for proper cytokinesis (Thompson et al., 2002; Wienke et al., 1999; Pelissier et al., 2003). However, mechanistic gaps still exist in our understanding of how DNM2 regulates cytokinesis.

Polo-like kinase 1 (Plk1) is a mitotic master regulator that is essential for several mitotic processes including mitotic entry, centrosome dynamics, polarity establishment, chromosome congression, chromosome alignment and cytokinesis (Petronczki et al., 2008; Archambault and Glover, 2009; Zitouni et al., 2014, Nishi et al., 2008). Importantly, Plk1 exhibits a dynamic localization pattern at different stages of mitosis. During early mitosis, Plk1 localizes to the centrosomes and the kinetochores. At anaphase, it re-localizes to the spindle midzone and at telophase, it localizes to the midbody (Golsteyn et al., 1995; Lee et al., 1995; Arnaud et al., 1998; Petronczki et al., 2008; Kishi et al., 2009; Schmucker and Sumara, 2014). The timely localization of Plk1 to various cellular components is essential for its different important roles during mitosis. Importantly, like DNM2, Plk1 is also required for proper cytokinesis (Petronczki et al., 2008; Archambault and Glover, 2009; Zitouni et al., 2014). Plk1 activity is required for recruitment of the RhoA guanine nucleotide exchange factor (GEF), Ect2, to the spindle midzone and equatorial cortex through phosphorylation of the centralspindlin component CYK4. Ect2, in turn, mediates RhoA activation to facilitate contractile ring assembly and cleavage furrow ingression, thereby ensuring proper cytokinesis (Brennan et al., 2007; Burkard et al., 2007; Petronczki et al., 2007; Santamaria et al., 2007; Petronczki et al., 2008; Burkard et al., 2009; Wolfe et al., 2009). Importantly, removal of Plk1 from the centrosomes is required for proper mitotic progression (Kishi et al., 2009). Although the kinase activity of Plk1 is thought to be required for Plk1 removal from the centrosomes (Kishi et al., 2009), the mechanism by which Plk1 is removed from the centrosomes remains unclear.

## RESULTS AND DISCUSSION

### *dyn-1 RNAi* embryos exhibit cytokinesis defects

To study the role of DYN-1 in cytokinesis, we depleted endogenous DYN-1 from *C. elegans* embryos expressing mCherry-SPD-2 (marks centrosomes) and GFP-histone (marks DNA) using *RNAi.* Importantly, our *RNAi* procedure successfully reproduced the cytokinesis failure phenotype that was previously reported by Thompson *et al*. upon *dyn-1 RNAi* in *C. elegans* (Thompson et al., 2002). While all of the control (S-adenosyl-methionine decarboxylase (*smd*-1)) *RNAi* treated embryos proceeded normally through cell division (n=28 embryos), 45% of the *dyn-1 RNAi* embryos failed cytokinesis (n=31 embryos) (Fisher’s exact test, p<0.0001) (Figure S1). Since our *RNAi* depletion was able to replicate the cytokinesis failure phenotype that was previously reported upon *dyn-1 RNAi* in *C. elegans* by another group (Thompson et al., 2002), we concluded that our depletion was successful. Therefore, we proceeded to investigate the downstream effects of DYN-1 depletion.

### *dyn-1* depletion results in abnormal metaphase spindle pole assembly

**Figure 1:**
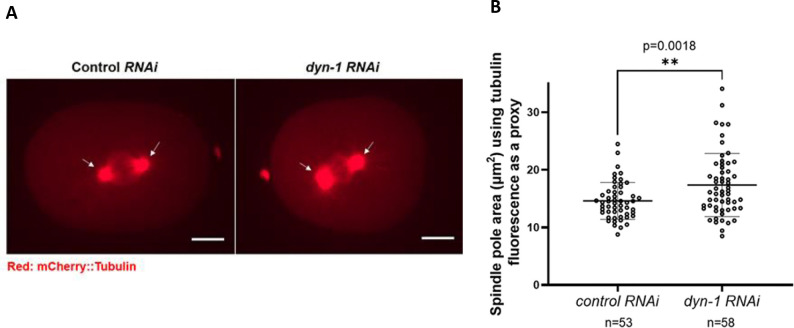
DYN-1 depletion increases spindle pole size. (A) Stills from live imaging of *C. elegans* embryos demonstrating that spindle pole size is increased upon *dyn-1 RNAi*. White arrows highlight spindle poles. Scale bar = 10 μm. (B) Quantification of spindle pole area in control and DYN-1-depleted embryos using mCherry-tubulin fluorescence as a proxy for the spindle poles. Each circle represents the area of a single spindle pole. n = number of spindle poles analyzed. Error bars represent the s.d; middle lines represent the mean. Unpaired two-tailed t-test, p=0.0018.

Interestingly, DYN-1 was shown to localize to the mitotic spindle at metaphase, suggesting a potential role in metaphase spindle assembly (Thompson et al., 2002). To determine the effect of DYN-1 depletion on metaphase spindle assembly, we depleted DYN-1 using *RNAi* and examined the effect of DYN-1 depletion on metaphase spindle assembly using mCherry-tubulin as a proxy (Figure 1). Upon measuring spindle pole area (as assessed by mCherry-tubulin fluorescence intensity), we determined that DYN-1-depleted embryos (n=58 spindle poles) exhibited a larger spindle pole area than control (*smd-1*) *RNAi* depleted embryos (n=53 spindle poles; unpaired two-tailed t-test, p=0.0018) (Figure 1). Specifically, the average spindle pole area of control *RNAi* treated embryos was 14.6 μm^2^ while that of *dyn-1 RNAi* treated embryos was 17.36 μm^2^. Thus, our data have identified a novel role for the endocytic protein DYN-1 in regulating metaphase spindle pole assembly. To explore this role further, we examined the mechanism by which DYN-1 might regulate spindle assembly. One protein that is an important regulator of mitotic spindle pole assembly is the kinase PLK-1. Furthermore, PLK-1 exhibits a partially overlapping localization pattern with DYN-1 during mitosis (Chase et al., 2000; Budirahardja and Gönczy, 2008; Martino et al., 2017; Thompson et al., 2002). Therefore, we questioned whether PLK-1 levels at the centrosomes were altered between control and *dyn-1 RNAi* treated embryos.

### DYN-1 depletion increases the levels of centrosome-associated PLK-1

**Figure 2:**
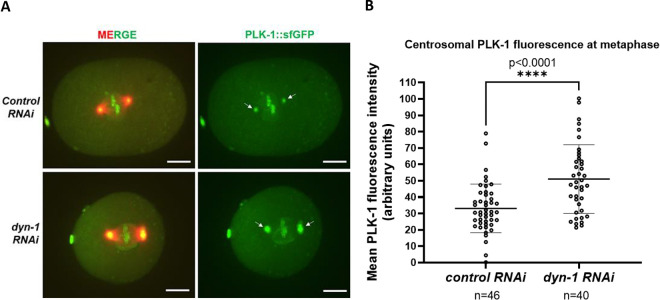
DYN-1 depletion increases centrosomal PLK-1-sfGFP localization. (A) Stills from live imaging assays of *C. elegans* embryos expressing PLK-1-sfGFP (green) and mCherry-tubulin (red) demonstrating that centrosomal localization of PLK-1-sfGFP is increased upon *dyn-1* depletion. White arrows represent centrosome-localized PLK-1. Scale bar = 10 μm. (B) Quantification of PLK-1-sfGFP fluorescence intensity upon control and *dyn-1 RNAi*. Each circle represents the PLK-1-sfGFP fluorescence intensity at a single centrosome. n = number of centrosomes analyzed. Error bars represent the s.d; middle lines represent the mean. Unpaired two-tailed t-test, p<0.0001.

To determine whether DYN-1 depletion affects the levels of centrosome-associated PLK-1, we created a new *C. elegans* strain expressing mCherry-Tubulin and CRISPR-tagged endogenous PLK-1-sfGFP (Martino et al., 2017) via genetic crossing. We then knocked down endogenous DYN-1 in this strain using *RNAi* and performed live imaging of control (*smd-1*) *RNAi* and *dyn-1 RNAi* treated embryos using spinning-disk confocal microscopy. Consistent with our hypothesis, our analysis revealed that PLK-1 levels at the centrosomes were elevated upon DYN-1 depletion (Figure 2). Specifically, we found that the average PLK-1 fluorescence intensity in DYN-1-depleted embryos was 51.02 arbitrary units (n=40 centrosomes). This was about 1.5 times higher than that of control (*smd-1) RNAi* treated embryos (average PLK-1 fluorescence intensity = 33.13 arbitrary units; n=46 centrosomes; unpaired two-tailed t-test, p<0.0001). These data indicate that DYN-1 normally acts to negatively regulate PLK-1 localization to the centrosomes through an as-yet unidentified mechanism. Therefore, in the absence of DYN-1, PLK-1 is no longer restricted from localizing to the centrosomes and consequently, its levels at the centrosomes increase. These increased levels of centrosome-associated PLK-1 upon DYN-1 depletion (Figure 2) are consistent with the enlarged spindle poles that we observed upon DYN-1 depletion (Figure 1). However, it is currently unclear whether DYN-1 regulates PLK-1 localization via vesicular trafficking or through an endocytosis-independent mechanism. Future studies will be directed towards further dissecting the molecular mechanism by which DYN-1 regulates PLK-1 localization.

### PLK-1 persists at the centrosomes following DYN-1 depletion in a subset of embryos during the later stages of mitosis

**Figure 3:**
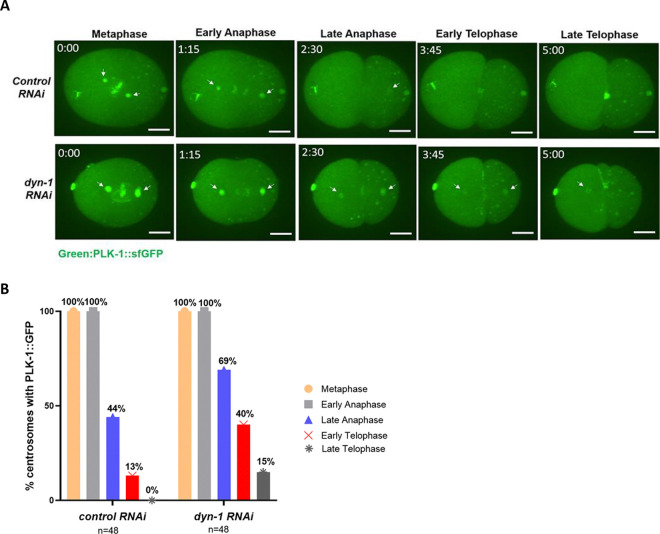
PLK-1-sfGFP persists at the centrosomes upon *dyn-1* knockdown (A) Stills from live imaging experiments of *C. elegans* embryos expressing PLK-1-sfGFP (green) demonstrating that PLK-1-sfGFP persists at the centrosomes upon *dyn-1* depletion. The metaphase timepoint was annotated as the zero timepoint. White arrows represent PLK-1-sfGFP at centrosomes. Scale bar = 10 μm. (B) Quantification of PLK-1-sfGFP localization at the centrosomes at different cell cycle stages. n = number of centrosomes analyzed.

PLK-1 exhibits a spatially and temporally regulated localization during different stages of mitosis (Chase et al., 2000; Budirahardja and Gönczy, 2008; Martino et al., 2017). PLK-1 primarily localizes to the centrosomes and to the kinetochores from prophase to metaphase. Upon anaphase onset, PLK-1 starts re-localizing to the spindle midzone. Finally, during telophase, PLK-1 completely disappears from the centrosomes and is recruited to the midbody to facilitate abscission (Chase et al., 2000; Budirahardja and Gönczy, 2008; Martino et al., 2017). During the course of our imaging experiments, we noticed several embryos where PLK-1 appeared to persist at the centrosomes (specifically at the pericentriolar material) during telophase (Figure 3A). To quantify this phenotype, we analyzed PLK-1 localization to the centrosomes at different cell cycle stages using maximum-intensity Z-projections of the time-lapse images from both control (*smd-1*) and *dyn-1 RNAi* treated embryos (Figure 3B). Centrosomes where PLK-1 fluorescence intensity was visually determined to be above that of the background cytoplasmic PLK-1-sfGFP fluorescence were scored as being positive for PLK-1. Our analysis revealed that in control embryos at late telophase, there was no PLK-1 detectable at any of the centrosomes analyzed (n=48 centrosomes) (Figure 3A and Figure 3B). In contrast, in 15% of the centrosomes from *dyn-1 RNAi* treated embryos (in 7 out of 48 centrosomes), PLK-1 was still detectable at the centrosomes at late telophase (Fisher’s exact test, p=0.0124) (Figure 3A and Figure 3B). We also noted a marked increase in the percentage of centrosomes with PLK-1 during early telophase upon DYN-1 depletion (Figure 3B). Specifically, while only 13% of the centrosomes (6 out of 48 centrosomes) from control embryos had PLK-1 associated with them during early telophase, 40% of the centrosomes (19 out of 48 centrosomes) from DYN-1-depleted embryos still had PLK-1 associated with them at this stage (Fisher’s exact test, p=0.0047) (Figure 3B). These data suggest that a pool of PLK-1 tends to persist at the centrosomes of DYN-1-depleted embryos during the later stages of mitosis. Since DYN-1 depletion results in an elevation of PLK-1 levels at the centrosomes during early mitosis, it is likely that these increased levels of PLK-1 make it difficult for PLK-1 to be effectively removed from the centrosomes during the later stages of mitosis in a subset of *dyn-1 RNAi* embryos.

### DYN-1-depleted embryos exhibit abnormal midbody formation

**Figure 4:**
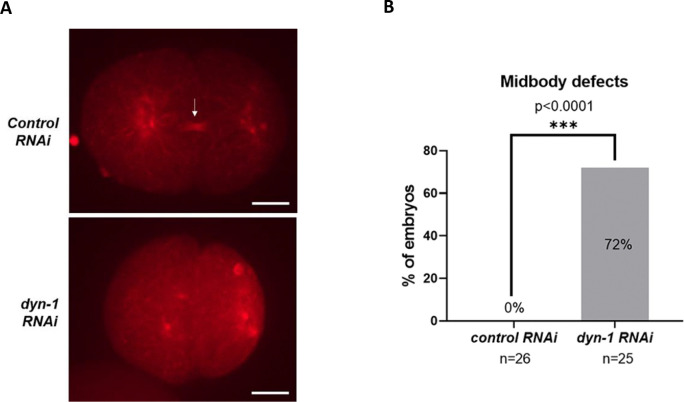
DYN-1 knockdown causes midbody defects. (A) Stills from live imaging of *C. elegans* embryos demonstrating that midbody assembly is defective upon DYN-1 depletion. Red: mCherry-tubulin, the white arrow represents a normal midbody. Scale bar = 10 μm. (B) Quantification of midbody defects observed upon *dyn-1* depletion. n= number of embryos analyzed. Fisher’s exact test, p<0.0001.

Since DYN-1 has a known role in cytokinesis and localizes to the midbody (Thompson et al., 2002), we analyzed midbody formation in DYN-1-depleted embryos using mCherry-tubulin fluorescence as a proxy for midbody assembly. We noted that all of the control *RNAi* treated embryos (n=26 embryos) exhibited a normal detectable bridge-like midbody during telophase as determined by intense tubulin fluorescence (Figure 4A (top panel, white arrow) and Figure 4B). In contrast, 72% of the *dyn-1 RNAi* treated embryos (18 out of 25 embryos) either exhibited a defective midbody or did not exhibit any detectable midbody during telophase (Figure 4A (bottom panel) and Figure 4B) (Fisher’s exact test, p<0.0001). In some of these instances, we observed that in DYN-1-depleted embryos, the midbody formed abnormally after telophase had already been completed and the embryo had entered the subsequent cell cycle. Together, these data suggest that DYN-1 function is required for proper midbody assembly.

### PLK-1 localization to the midbody is defective in embryos depleted of DYN-1

**Figure 5:**
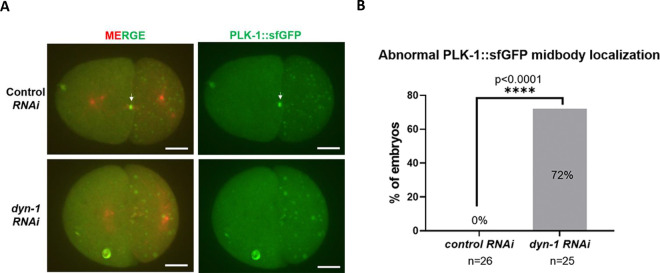
PLK-1-sfGFP fails to localize correctly to the midbody upon *dyn-1 RNAi*. (A) Stills from live imaging of *C. elegans* embryos expressing PLK-1-sfGFP (green) and mCherry-tubulin (red) demonstrating that the midbody localization of PLK-1 is perturbed upon *dyn-1* depletion. The white arrow represents a normal PLK-1 localization at the midbody. Scale bar = 10 μm. (B) Quantification of PLK-1-sfGFP midbody localization upon *dyn-1 RNAi*. n = number of embryos analyzed. Fisher’s exact test, p<0.0001.

Since our previous data demonstrated that PLK-1 continued to localize to the centrosomes at the later mitotic stages in a subset of DYN-1-depleted embryos (Figure 3), we hypothesized that this may correlate with an abnormal localization of PLK-1 to the midbody during late mitosis. Indeed, time-lapse live imaging revealed that PLK-1-sfGFP localization to the midbody was defective in DYN-1 depleted embryos (Figure 5). Specifically, we found that while all of the control embryos (n=26 embryos) exhibited a stereotypical localization of PLK-1-sfGFP at the center of the midbody, in about 72% of the DYN-1-depleted embryos (18 out of 25 embryos), PLK-1-sfGFP failed to localize correctly to the midbody (Figure 5) (Fisher’s exact test, p<0.0001). As reported previously, in some severe cases, the cleavage furrow failed to form completely upon DYN-1 depletion (Thompson et al., 2002). In these cases, we observed that PLK-1-sfGFP localization was severely defective during both anaphase and telophase (Supplementary Movie S1). Notably, PLK-1-sfGFP failed to localize correctly to the midbody in 17 out of the 18 embryos that were quantified as having midbody defects in Figure 4. These data indicate that proper PLK-1 localization to the midbody is likely a prerequisite for normal midbody assembly.

### Total protein levels of Plk1 are not increased upon DNM2 depletion in HeLa cells

Since we observed increased PLK-1 levels at the centrosomes upon DYN-1 depletion (Figure 2), we asked whether this was occurring due to an overall increase in total PLK-1 levels or due to a specific enhancement of PLK-1-sfGFP localization to centrosomes. To answer this question, we performed western blot analysis of *C. elegans* whole worm lysates from control and *dyn-1 RNAi* worms and probed them for PLK-1-sfGFP. Unfortunately, we could not detect any PLK-1-sfGFP expression in our lysates, possibly due to its low abundance in whole worm lysates (data not shown). Since both PLK-1 and DYN-1 proteins are conserved between humans and *C. elegans* (Clustal Omega), we turned to HeLa cells to answer this question. We were able to successfully knockdown DNM2 (human DYN-1 homolog) in HeLa cells (Figure S2A). The DNM2 knockdown efficiency in our experiments ranged from ~81% to 95.4% (Figure S2B). Normalized Plk1 expression levels measured from four independent experiments did not reveal a statistically significant increase in Plk1 levels upon DNM2 depletion (Figure S2C) (unpaired two-tailed t-test with Welch’s correction, p=0.4516). These data support the notion that the total cellular levels of Plk1 are likely not increased upon DNM2 depletion.

In summary, our studies have identified a new role for the endocytic protein DYN-1 in regulating metaphase spindle assembly and PLK-1 localization during mitosis. Specifically, we find that upon DYN-1 depletion, PLK-1 accumulates at the centrosomes in metaphase and this is associated with aberrant metaphase spindle pole assembly. Furthermore, while PLK-1 is normally removed from the centrosomes during late mitosis, upon DYN-1 depletion, we find that PLK-1 persists at the centrosomes during the later stages of mitosis. We hypothesize that a failure to remove PLK-1 from the centrosomes in a timely manner likely causes midbody defects and results in cytokinesis failure in DYN-1-depleted embryos.

Moving forward, a detailed mechanistic analysis will be carried out to further investigate the cross-talk between DYN-1 and PLK-1. Specifically, we will utilize mutants such as *dyn-1(ky51ts)* to determine if the vesicular trafficking function of DYN-1 is required for this phenotype (Clark et al., 1997; Grant and Hirsh 1999). Future experiments will be also designed to test if DYN-1 and PLK-1 physically interact or if perturbing the functions of other endocytic proteins phenocopies the DYN-1 depletion phenotype. We will also determine whether the localization of key cytokinesis regulators (e.g. CYK-4, NOP-1, NMY-2, ANI-1, RHO-1, ECT-2) is altered upon DYN-1 depletion. Rescue experiments involving partial knockdown of PLK-1 in *dyn-1 RNAi* embryos could potentially clarify whether elevated centrosomal PLK-1 levels contribute to the observed mitotic and cytokinetic defects of DYN-1 depletion. However, in our experience, achieving consistent and titratable knockdown of PLK-1 using *RNAi* by feeding has proven challenging. As a result, this experiment was not pursued in the present study. As an alternative, future studies will utilize the temperature-sensitive *plk-1(or683ts)* mutant to achieve partial loss of PLK-1 function under semi-permissive conditions (O’Rourke et al., 2011). This allele was shown to remove PLK-1 from centrosomes in five hours at the restrictive temperature of 25 °C (Martino et al., 2017). To test whether reducing PLK-1 levels can rescue the cytokinesis defects of *dyn-1*–depleted embryos, we will need to carefully titrate the inactivation window to achieve centrosomal PLK-1 levels compatible with normal cell division. While promising, this strategy is technically challenging, as it requires precise timing of embryo imaging within a narrow window to achieve the optimal level of PLK-1 reduction. Despite these constraints, this approach offers a powerful means to directly test whether elevated PLK-1 at centrosomes underlies the mitotic and cytokinetic phenotypes observed upon DYN-1 depletion.

## MATERIALS AND METHODS

### *C. elegans* strains growth and maintenance

All *C. elegans* strains described in this study were grown and maintained using standard techniques on OP50 seeded Modified Youngren’s Only Bacto-peptone (MYOB) agar plates at 20°C (the strains were shifted to other assay-specific temperatures as needed).

### *C. elegans* strains and genetic crosses

The wild-type N2 strain and the OD2425 strain expressing PLK-1-sfGFP (*plk-1(lt18[plk-1::sfGFP]::loxp) III)* (Martino et al., 2017) were obtained from the *Caenorhabditis Genetics Center* (CGC). The OC908 strain (*bsSi30[pCW9: unc-119(+) pcdk-11.2::sfgfp::his-58::cdk-11.2 3’ UTR] II; bsIs20[pNP99: unc-119(+) tbb-1p::mCherry::tbb-2::tbb-2 3’-UTR]; bsIs2[pCK5.5: Ppie-1::gfp::spd-2]*) and the OC779 strain (*bsSi15[pKO109: spd-2p-spd-2::mCherry::spd-2 3’-utr, unc-119(+)] I; bsSi30[pCW9: unc-119(+) pcdk-11.2::sfgfp::his-58::cdk-11.2 3’ utr] II; unc-119(ed3) III*) were obtained from Dr. Kevin O’Connell’s laboratory at NIDDK, NIH. The mCherry–tubulin–expressing strain IYR028 (*bsIs20[pNP99: unc-119(+) tbb-1p::mCherry::tbb-2::tbb-2 3’-UTR]*) was generated in our laboratory by crossing OC908 hermaphrodites with N2 males and isolating progeny carrying only the mCherry-tubulin transgene. Following this, mCherry-tubulin expressing males were generated from the IYR028 strain by heat shock at 30°C for 6 hours. These mCherry-tubulin males from the IYR028 strain were then crossed with PLK-1-sfGFP expressing hermaphrodites from the OD2425 strain to generate the IYR038 strain (*bsIs20[pNP99: unc-119(+) tbb-1p::mCherry::tbb-2::tbb-2 3’-UTR]; plk-1(lt18[plk-1::sGFP]::loxP) III)* which was homozygous for both mCherry-Tubulin and PLK-1-sfGFP. This IYR038 strain was used for all but one of the imaging experiments described in this study. The experiment involving the analysis of cytokinesis failure utilized the OC779 strain expressing mCherry SPD-2 and GFP-histone.

### *RNAi* by feeding

HT115 bacteria expressing the *dyn-1 RNAi* construct were purchased from Horizon Discovery Ltd. (Lafayette, CO) while HT115 bacteria expressing the *smd-1 RNAi* construct (control) were obtained from Dr. Kevin O’Connell’s laboratory (NIDDK, Bethesda, MD). HT115 *E. coli* transformed with either control (*smd-1)* or *dyn-1 RNAi* plasmids were grown on MYOB plates supplemented with 50 μg/ml carbenicillin and 2 mM Isopropyl β- d-1-thiogalactopyranoside (IPTG) as described previously (Kamath and Ahringer J 2003; Iyer et al., 2022). Briefly, L4-stage *C. elegans* from either the IYR038 or the OC779 strains were transferred onto the respective *RNAi* plates and incubated at 25 °C for 24 to 36 hours. Gravid adults from these plates were then dissected to release their embryos and 1-cell stage embryos were analyzed by confocal live imaging.

### *C. elegans* embryo live imaging using a spinning-disk confocal microscope

*C. elegans* embryos obtained from dissected *smd-1* and *dyn-1 RNAi* treated worms were imaged using an Olympus Yokagawa X1 spinning-disk confocal microscope as described previously (Bergwell et al., 2023) with a few alterations. Timelapse imaging was performed using the cellSens software (Olympus America Inc.) by taking 1.5 μm Z-stacks every ~75 seconds at a 60X magnification with a 1.42 numerical aperture (NA) objective using a Prime 95B CMOS camera. To enable quantitative analysis, all images were captured at the same laser intensity (50% laser intensity for both 561 nm and 488 nm channels) and exposure time (700 msec exposure time for both 561 nm and 488 nm channels). Maximum intensity Z-projections were obtained using the cellSens software and converted to a .png format for all representative images and analyses. All brightness and contrast adjustments were applied equally for both the control and *dyn-1 RNAi* representative images to enable better image presentation and viewing.

### Cytokinesis failure quantification

To score cytokinesis failure, the development of each embryo was monitored from the 1-cell stage to the 4-cell stage. If the embryonic nuclei coalesced back to set up a multipolar spindle at the 2-cell stage, the embryo was scored as having failed cytokinesis.

### Spindle pole area measurement

To measure spindle pole area, the cellSens software (Olympus America Inc.) was used to obtain maximum intensity Z-projections of the original .vsi files. These Z-projections were saved in a .png format with the fixed scaling (left:80 and right:400) for all images and further analyzed using the Nikon NIS-Elements software (Nikon Instruments Inc.). The calibration on the NIS-Elements software was set to 1 pixel = 0.183 μm. The thresholding tool in NIS-Elements was used to define and compute the spindle pole area (in μm²) (intense mCherry-tubulin fluorescence demarcated each spindle pole). Only regions with clearly defined spindle poles were included in the analysis. Embryos in which spindle poles could not be reliably distinguished were excluded.

### PLK-1 metaphase centrosome intensity measurements

In our time-lapse imaging experiments, the stage immediately prior to anaphase onset (~75 seconds before anaphase) was designated as metaphase. Notably, there was some embryo-to-embryo variability in chromosome alignment at this stage; in some embryos, the chromosomes were centrally aligned, while in others, they were still in the process of aligning. To quantify PLK-1–sfGFP fluorescence at the centrosomes, .avi files of maximum intensity Z-projections of metaphase embryos were analyzed using Nikon NIS-Elements software (Nikon Instruments, Inc.), with image calibration set to 1 pixel = 0.183333 μm. A region of interest (ROI) measuring 13.81 μm² was drawn around each centrosome and within the cytoplasm of the same embryo. Cytoplasmic PLK-1–sfGFP intensity was then subtracted from centrosomal PLK-1-sfGFP intensity to calculate the mean PLK-1-sfGFP fluorescence intensity, reported in arbitrary units. Embryos with inaccurate PLK-1-sfGFP fluorescence due to debris on top of the embryo affecting maximum intensity Z-projections were excluded from our analysis. Embryos where the centrosomal PLK-1–sfGFP signal could not be clearly distinguished from the chromosomal PLK-1-sfGFP signal were also excluded from our analysis.

### Analysis of PLK-1 persistence at centrosomes

To analyze PLK-1-sfGFP persistence at centrosomes, maximum intensity Z-projections of each timelapse sequence were visually analyzed for the presence or the absence of PLK-1-sfGFP at the centrosomes. Centrosomes where the fluorescence intensity of PLK-1-sfGFP was visually determined to be greater than the cytoplasmic background PLK-1-sfGFP fluorescence were scored as being positive for PLK-1-sfGFP. The cell cycle stages were classified as follows: metaphase: the chromosomes were aligning at the center of the cell and a diamond-shaped spindle was formed, early anaphase: ~75 seconds after metaphase when the chromosomes start to separate, late anaphase: ~75 seconds after early anaphase, early telophase: ~75 seconds after late anaphase and late telophase: ~75 seconds after early telophase.

### Quantification of midbody abnormalities

Maximum intensity Z-projections of the 561 (red) channel from the imaged IYR038 strain were analyzed for midbody defects. Midbody assembly was analyzed at either 300 or 375 seconds after metaphase onset. A normal midbody was classified as a structure where an intense tubulin bridge was clearly visible at the abscission area close to the center of the embryo. An abnormal midbody was defined as one where either no intense tubulin bridge connecting the two daughter cells was detected or where a structure resembling the midbody formed after the cell had already entered the next cell cycle.

### Quantification of PLK-1 midbody localization defects

Maximum intensity Z-projections were used to quantify PLK-1-sfGFP midbody localization defects. PLK-1-sfGFP localization to the midbody was analyzed at either 300 or 375 seconds after metaphase onset. Embryos where a bright PLK-1-sfGFP spot could be detected at the abscission point during early to late telophase were classified as exhibiting a normal PLK-1 midbody localization. Embryos where PLK-1-sfGFP exhibited a scattered localization at the cleavage furrow during early to late telophase were classified as exhibiting an abnormal PLK-1 localization.

### Statistical analysis of *C. elegans* studies

Graphpad prism 10.5 (GraphPad Software, Inc., San Diego, CA) was used to perform statistical analyses of all the data. For all the data, error bars represent the standard deviation (s.d.) above and below the mean and the middle lines represent the mean. *p*-values were rounded to two decimal places and considered significant if *p*<0.05. A two-tailed unpaired t-test was used to analyze the spindle pole area and the PLK-1-sfGFP fluorescence intensity data. A two-tailed unpaired t-test with Welch’s correction was used to analyze band intensities from western blotting. A Fisher’s exact test was used to analyze the cytokinesis defects, PLK-1-sfGFP persistence at the centrosomes, defects in midbody formation and defects in PLK-1-sfGFP localization to the midbody.

### Cell culture and siRNA transfections

HeLa cells were maintained by culturing them in Dulbecco’s Modified Eagle Medium (DMEM) (Gibco, Catalog #11–995-040) with 10% fetal bovine serum (FBS) (Corning, Catalog #MT35010CV) and 1% penicillin/streptomycin (1% P/S) (Gibco, Catalog #15–140-122) in a 37^°^C incubator with 5% CO_2_. For DNM2 siRNA experiments, HeLa cells were seeded at a density of up to 2.4X10^5^ cells per well (20 to 30% confluence) into each well of a 6-well plate and allowed to adhere to the plate for 24 hours prior to transfection. The cells were rinsed twice with DPBS (Gibco, Catalog # 14040–117) prior to transfection. Control and DNM2 siRNA constructs were obtained from Santa Cruz Biotechnology Inc. (Control siRNA Catalog # sc-37007; DNM2 siRNA Catalog # sc-35236). The siRNA constructs were transfected into HeLa cells using Lipofectamine LTX reagent (Thermo Fisher Scientific, Inc., Catalog # 15–338-030) in 1 ml of reduced serum OPTI-MEM medium (Gibco, Catalog # 31985070) according to the manufacturer’s instructions. After six hours, 1 ml of DMEM containing 20% FBS and 2% P/S was added to the transfected cells. After 24 hours, the media containing the transfection reagent was removed and replaced with fresh DMEM with 10%FBS and 1% P/S. The cells were allowed to grow for 48 hours and then lysed with RIPA buffer (Santa Cruz Biotechnology Inc, Catalog # sc-24948A) containing 1 mM phenylmethylsulfonyl fluoride (PMSF) (Thermo Fisher Scientific, Inc., Catalog # 36978) and a protease inhibitor cocktail (Pierce Protease Inhibitor Tablets, EDTA-free, Catalog #A32965). Lysate protein concentrations were measured using a Bradford assay (Thermo Scientific Inc., Catalog # 23200).

### Western blotting

The HeLa cell lysates from control and DNM2 siRNA treated cells were subjected to western blot analysis using the Bio-Rad wet transfer method. Briefly, approximately 20 micrograms of protein from each sample were loaded onto a well of an SDS-PAGE gel (Genscript Corporation, Catalog # M00656). The gel was run until adequate protein separation was achieved. Following electrophoresis, the proteins were subjected to an overnight transfer onto a 0.2 μm nitrocellulose membrane (Thermo Fisher Scientific Inc., Catalog # LC2000) for 16 hours at 30V using the Towbin buffer (25 mM Tris base, 192 mM Glycine, 20% (v/v) Methanol) at 4°C. The membrane was then stained with the Ponceau S solution (Fisher Scientific Inc. Catalog # A40000279) to ensure good protein transfer. Following this, the membranes were blocked with blocking buffer (1X Tris-buffered saline (TBS) (0.05 M Tris, 0.15 M sodium chloride, pH=7.4), 0.2% gelatin from cold water fish skin (Sigma-Aldrich Inc., Catalog # G7041), 0.1% Tween 20 (Thermo Scientific Inc., Catalog # J20605-AP) and 0.02% sodium azide (RPI Research Products, Catalog # S24080–250)) at room temperature for 30 minutes. Once blocked, the membranes were probed with a rabbit polyclonal anti-DNM2 antibody at a 1:1000 dilution (Abcam Inc., Catalog # ab3457), a mouse monoclonal anti-tubulin antibody at a 1:100 dilution (Santa Cruz Biotechnology Inc., Catalog # sc-32293), a mouse monoclonal anti-Plk1 antibody at a 1:100 dilution (Santa Cruz Biotechnology Inc., Catalog # sc-17783) for 2 hours at room temperature. Subsequently, the membrane was washed 3 times with 1X TBS supplemented with 0.1% Tween-20 (1XTBST) and incubated with the corresponding secondary antibodies (IRDye 680RD goat anti-rabbit IgG secondary antibody (LI-COR Biotech, LLC, Catalog # 926–68071) and IRDye 800CW donkey anti-mouse IgG secondary antibody (LI-COR Biotech, LLC, Catalog # 926–32212)) at a 1:10,000 dilution for 1 hour at room temperature. The membrane was washed again 3 times using 1XTBST and imaged using the LI-COR Odyssey Fc imaging system (LI-COR Biotech, LLC).

### Western blot intensity analysis

The band intensities for the different proteins were quantified using the LI-COR Image Studio software (LI-COR Biotech LLC) and normalized with respect to the loading control (tubulin). Band intensities from four independent western blot experiments were analyzed and plotted.

## Supplementary Material

Supplement 1

Supplement 2

Supplement 3

## SIGNIFICANCE STATEMENT

Dynamin is primarily known for its function in endocytosis, but its involvement in mitosis is not well defined. This study shows that dynamin helps organize the mitotic spindle and ensures proper localization of the master mitotic kinase PLK-1 in *C. elegans* embryos. These findings reveal a previously unrecognized role for dynamin in mitosis and suggest new links between membrane dynamics and the cell division machinery.
