# Supplementary material for: Dynamin regulates PLK-1 localization and spindle pole assembly during mitosis
Source: bioRxiv. 2025 Jul 21:2025.07.21.665896. Preprint. [Version 1] doi: 10.1101/2025.07.21.665896 (PMC12330643; doi:10.1101/2025.07.21.665896)
Supplement: Supplement 1 [file media-1.pdf]

Figure S1

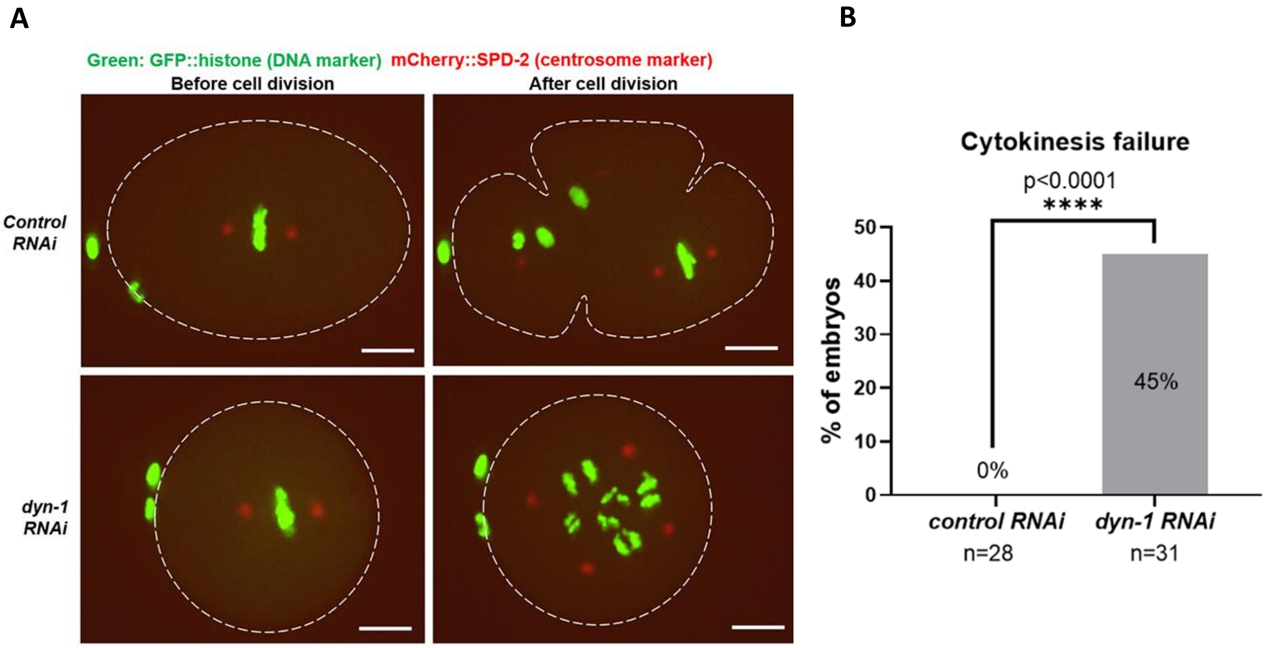

Figure S1: DYN-1 depletion causes cytokinesis failure. (A) Stills from live imaging of *C. elegans* embryos expressing GFP-Histone (green) and mCherry-SPD-2 (red) demonstrating that as reported previously (Thompson *et al.,* 2002), cytokinesis fails upon DYN-1 depletion. Scale bar = 10 µm. (B) Quantification of cytokinesis defects. n = number of embryos analyzed. Fisher's exact test, $p<0.0001$.

Figure S2

A

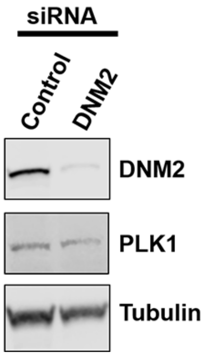

B

Experiment #	DNM2 knockdown efficiency
Experiment 1	95.4%
Experiment 2	91.6%
Experiment 3	80.6%
Experiment 4	83.7%

C

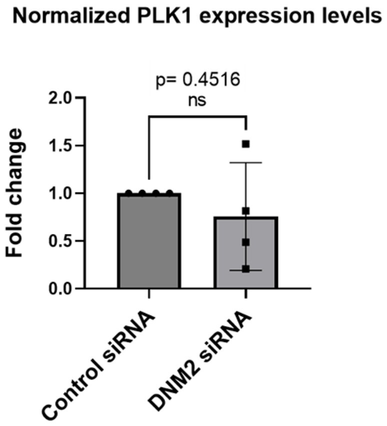

Figure S2: Total PLK1 levels do not increase upon DNM2 depletion in HeLa cells. (A) Representative western blot demonstrating the successful knockdown of human DNM2 in HeLa cells. (B) Table representing DNM2 knockdown efficiencies for four independent experiments. (C) Quantification of PLK1 band intensities upon DNM2 siRNA. Unpaired two-tailed t-test with Welch's correction, p=0.4516.
