# Supplementary material for: Dynamin regulates PLK-1 localization and spindle pole assembly during mitosis
Source: bioRxiv. 2025 Jul 21:2025.07.21.665896. Preprint. [Version 1] doi: 10.1101/2025.07.21.665896 (PMC12330643; doi:10.1101/2025.07.21.665896)
Supplement: Supplement 2 [file media-2.pdf]

Supplemental Video S1. *dyn-1 RNAi* embryo expressing mCherry-tubulin and PLK-1-sfGFP exhibiting a severe PLK-1 localization defect.
